# Supplementary material for: Genome Sequencing of Consanguineous Family Implicates Ubiquitin-Specific Protease 53 (USP53) Variant in Psychosis/Schizophrenia: Wild-Type Expression in Murine Hippocampal CA 1–3 and Granular Dentate with AMPA Synapse Interactions
Source: Genes (Basel). 2023 Oct 9;14(10):1921. doi: 10.3390/genes14101921 (PMC10606770; doi:10.3390/genes14101921)
Supplement: Supplementary file 1 [file genes-14-01921-s001.zip › genes-2602017-supplementary.pdf]

Supplementary Information

Genome sequencing of consanguineous family implicates ubiquitin specific protease (*USP53*) variant in psychosis/schizophrenia: USP53 wild type expression in murine hippocampal CA 1-3 and granular dentate with AMPA synapse Interactions

Ambreen Kanwal, Sohail A. Sheikh, Faiza Aslam, Samina Yaseen, Zachary Beetham, Nathan Pankratz, Sadaf Naz, José V. Pardo

Supplementary Information

Supplementary Figures	p. 3-6
Supplementary Tables	p. 7-20
Supplementary Materials and Methods	p. 21-29
Supplementary References	p. 30

Supplementary Materials

Supplementary materials are published online alongside the manuscript (pdf).

Supplementary Figure 1. WGS/GATK/Genvisis Analysis.

Supplementary Figure 2. Other regions of homozygosity for pedigree PSY01.

Supplementary Figure 3. Samplot of structural variant signals vs. chromosome 4 location.

Supplementary Figure 4. Additional Sanger sequences for Figure 1.

Supplementary Table 1. Typical parameters from whole exome sequencing.

Supplementary Table 2. Primer sequences for amplification.

Supplementary Table 3. Rare homozygous variants in affected individuals and heterozygous in parent.

Supplementary Table 4. Genes of interest.

Supplementary Table 5. Autosomal dominant model: missense and LOF from genome sequencing data.

Supplementary Table 6. Autosomal recessive model: missense and LOF from genome sequencing data (note: USP53 was excluded because GDI > 75 percentile threshold).

Supplementary Table 7. Antibodies used & concentrations.

Supplementary Table 8. Antibodies used in immunohistochemistry and western blots.

.

Supplementary Figure 1. WGS/GATK & Genvisis Analysis

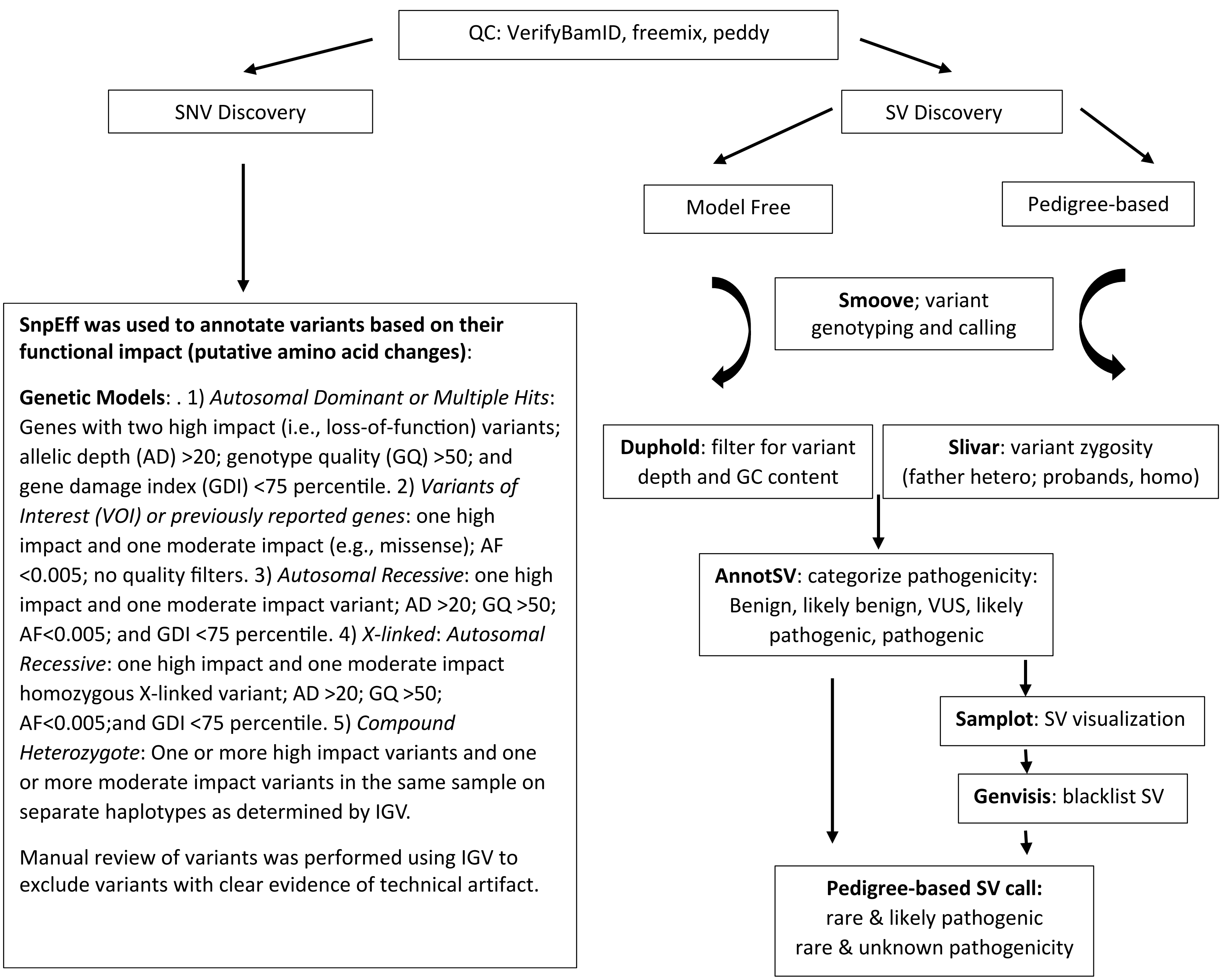

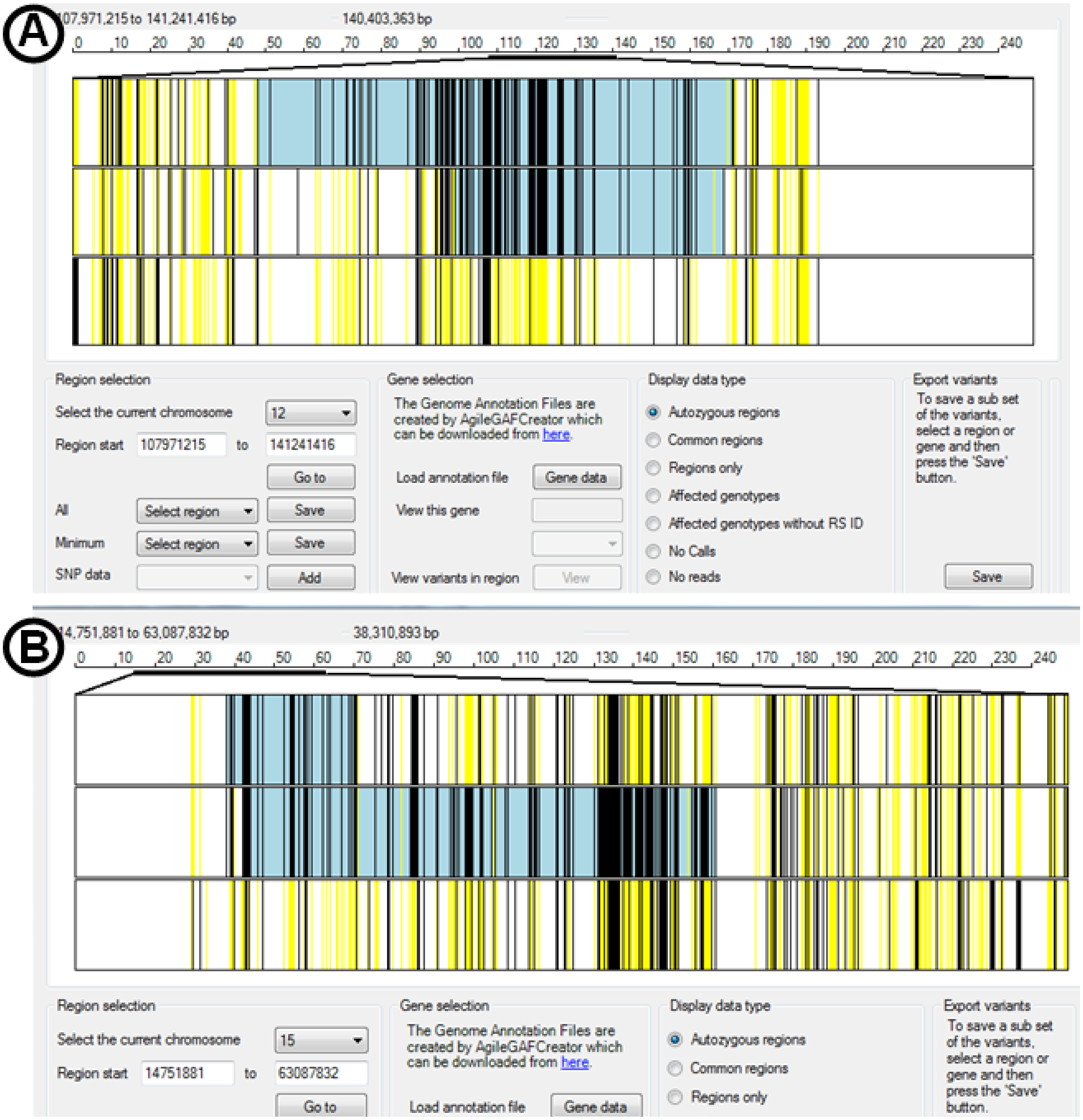

Supplementary Figure21. Other regions of homozygosity for Family PSY01.
Based on homozygosity mapping, only 7 rare homozygous variants surfaced in the affected individuals but were heterozygous in the parent.
A) A region of homozygosity from exome data was displayed at 118-130 Mb on chr12 for two affected individuals of family PSY01 that was heterozygous in the parent. However, none of the variants located in this region were likely to be pathogenic (please see Supplementary Table 2). B) A region of homozygosity from exome data was displayed at 21-33 Mb on chr15 for two affected individuals of family PSY01 that was heterozygous in the parent. However, no rare exonic or intronic variant was detected in the exome data (Please see Supplementary Table 1).

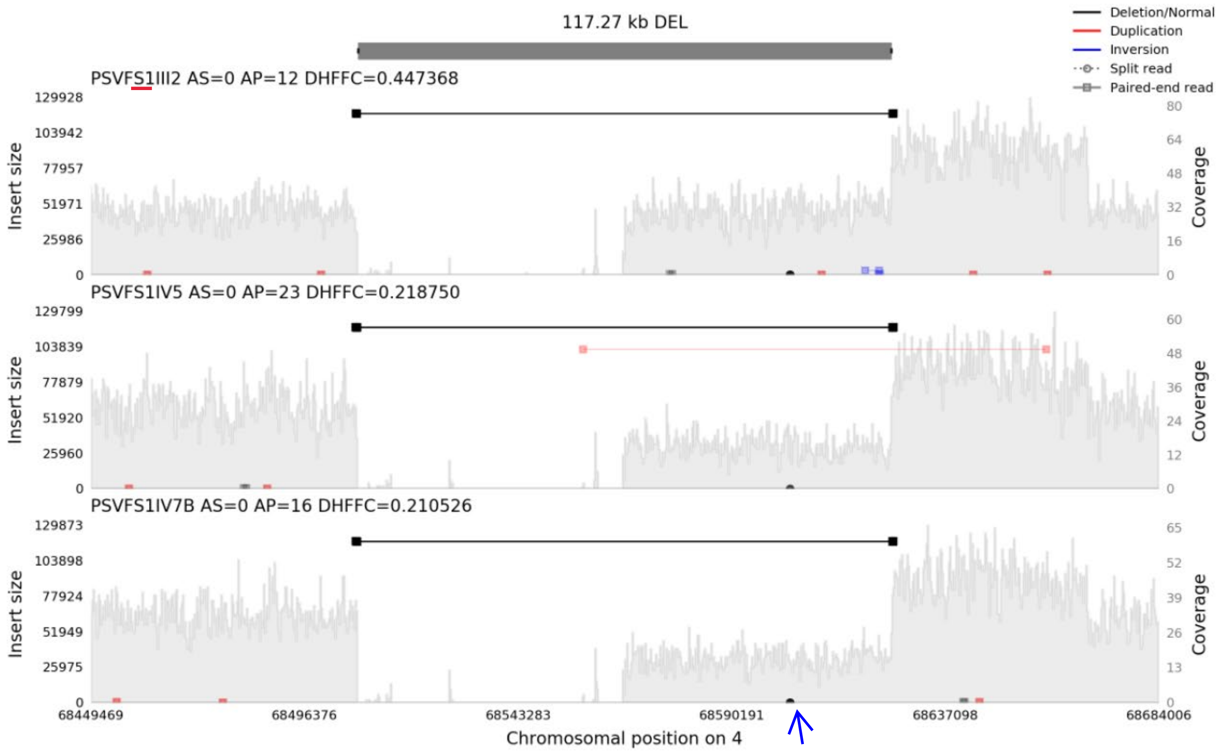

Supplementary Figure 3[illegible] . Samplot of structural variant signals vs. chromosome 4 location. Plot shows insert size and coverage on the abscissas with the location of the 304 bp deletion (arrow) and 117 kb deletion for father and both affected daughters. DHFFC, duphold flank fold-change.

Supplementary Figure 4. Additional Sanger sequences (for Figure 1)

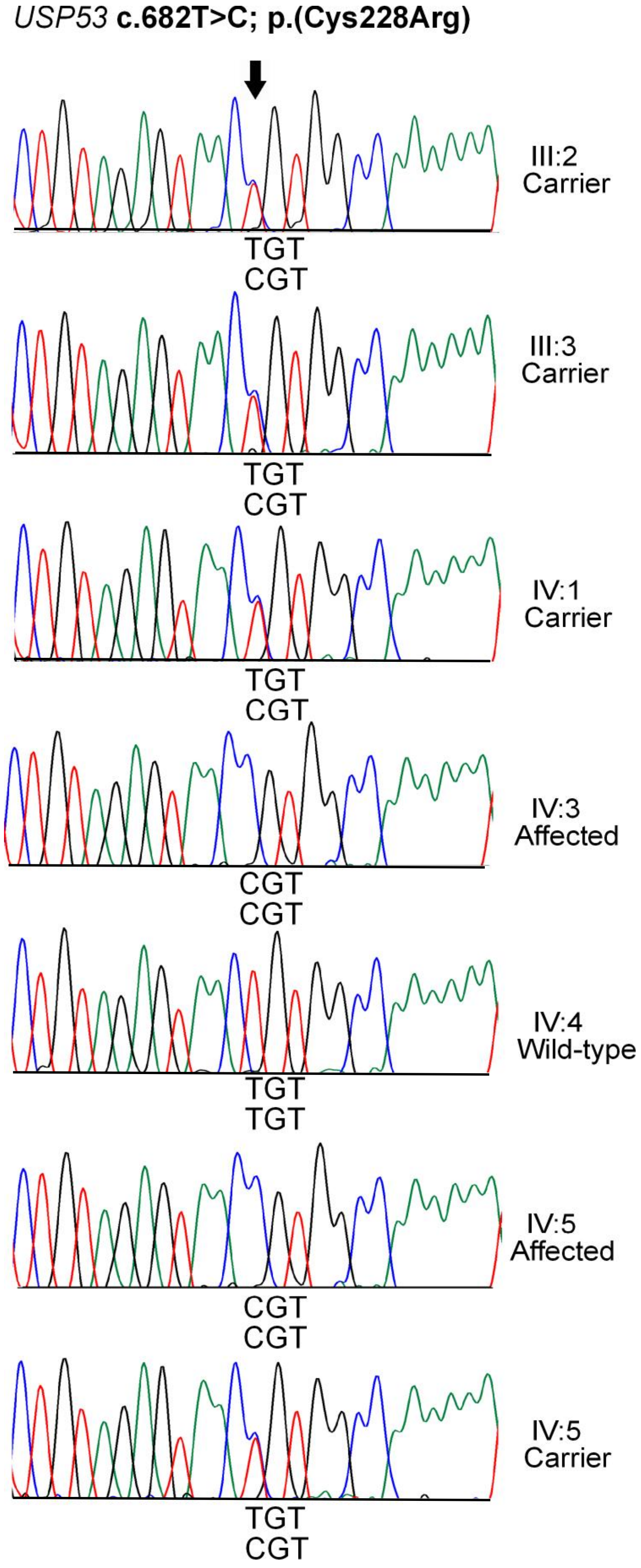

Supplementary Table 1. Typical WES output parameters.

Parameter	Value
Total reads	$4(10)^{7}$
Total yield bp	$6(10)^{9}$
Average read length bp	149
Target regions bp	5(10)
% target coverage	97
Average target depth	75
% On-target reads	76
% exome covered > once	99.6
# SNPs	82K
# missense variants	10.6K
# frame shift variants	290
# stop loss	36
# stop gain	83
mean depth target regions	65
# Indels	11K

Supplementary Table 2. Primers for amplification.

Primer name	Sequence	Product (size bp)	*Comment
USP53_F<br>USP53_R	TGACTTCAAGAGTTTTATTTCTGC<br>AGGCATTCCAGTTAGCCTGA	525	For amplification and Sanger sequencing
USP53_OF<br>USP53_OR	TTGATGAATGATGAATGATACTCTTTTCTCATC<br>GAAGAATTTGAAAATAAAAGGGAAGGGAATG	505	For use in Tetra-ARMS PCR, constant product
USP53_MF	TTTAAAACTTTTATGTTTTTCTGTAGAGTA**C**CC	223	Yields product with USP53_OR only from the mutant allele
USP53_NR	ACGGCGAATTTTTATTTTTTGGCC**C**CA	347	Yields product with USP53_OF only from the wild-type allele

Primers for PCR amplification and Sanger sequencing were designed using primer3 (https://bioinfo.ut.ee/primer3-0.4.0/). PCR reactions were performed under standard conditions and purified products were sequenced using BigDye Terminators v3.1. Primers for Tetra-Primers ARMS PCR were designed using primer1 (http://primer1.soton.ac.uk/primer1.html). All four primers were used in one reaction of 10μl by using 1μM each (USP53_OF - outer forward primer and USP53_OR - outer reverse primer) & 10μM each (allele specific primers, mutant specific-USP53_MF and wild-type specific - USP53_NR) primers. Nucleotides in bold & underlined in the USP53_MF and USP53_NR primers indicate bases which were deliberately mismatched to avoid non-specific amplification by these primers of the wild-type or mutant alleles, respectively.

Supplementary Table 3. Rare homozygous variants in affected individuals and heterozygous in parent.

Gene	*Position	Trancript	ΔcDNA	Effect	gnomAD MAF	**Prediction	Comment
PRSS12	Chr4:119203363	NM_003619.4	c.2356A>G	p.Ile786Val	0.00001193‡	Benign; ACMG variant of unknown significance	The amino acid is not conserved in evolution. Mouse and xenopus othologs have valine in this position. Mean allele frequency (MAF) = 0.003
USP53	Chr4:120181668	NM_019050.2	c.682T>C	p.Cys228Arg	0.00002499‡	Deleterious; ACMG variant of unknown significance	Affected amino acid is conserved in evolution, variant segregates with the phenotype. The variant was absent from 600 ethnically matched chromosomes of unrelated individuals
BBS12	Chr4:123664553	NM_152618.3	c.1506C>T	p.Ala502Ala	0.0002585‡	None; ACMG likely benign; ClinVAR VCV000166729.6 likely benign	Synonymous variant. MAF = 0.01, including one homozygous individual.
LRRC43	Chr12:122685380	NM_001098.519.2	c.1708G>T	p.Val570Leu	0.0001987°	Benign; ACMG likely benign	The amino acid is not conserved in evolution. Canine ortholog has Leucine in this position.
DNAH10	Chr12:124323125	NM_207437.3	c.4671C>T	p.Tyr1557Tyr	0.001147°	Benign; ACMG likely benign	Synonymous variant, MAF = 0.003.
NCOR2	Chr12:124821723	NM_006312.6	c.5691C>T	p.Ser1897Ser	0.00005492‡	None; ACMG variant of unknown significance	Synonymous variant, Neither the nucleotide nor the affected amino acid is conserved in evolution.
DHX37	Chr12:125450356	NM_032656.4	c.1593G>A	p.Val531Val	0.00005992‡	None; ACMG variant of unknown significance	Synonymous variant. Neither the nucleotide nor the affected amino acid is conserved in evolution. MAF = 0.003.

*Variant position according to Human Feb.2009 (GRCh 37/hg19) Assembly. ** Pathogenicity predictions from multiple software as well as from ACMG classification. ACMG = American College of Medical Genetics and Genomics interpretation; gnomAD = genome Aggregation Database; MAF = mean allele frequency; ‡, non-homozygous; °, homozygous.

Supplementary Table 4. Genes of interest.

Case	Gene	Variant	Locus and Reference Allele	Allele 1 and Allele 2	GQ	AD	Impact	AF	CADD	GDI
IV:3	*USP53*	p.Cys228Arg	4:119260513-TRUE	[C, C]	81	[0, 27]	missense_variant	0.0004	27.6	82.83479
IV:5	*USP53*	p.Cys228Arg	4:119260513-TRUE	[C, C]	99	[0, 33]	missense_variant	0.0004	27.6	82.83479

GQ, genotype quality; AD, allelic depth; AF, allele frequency; NMD, nonsense mediated decay; CADD, Combined Annotation Dependent Depletion; GDI, Gene Damage Index

Supplementary Table 5. Autosomal dominant model: missense and LOF from genome sequencing data.

Case	Gene	Variant	Locus and Reference Allele	Allele 1 and Allele 2	GQ	AD	Impact	AF	CADD	GDI
IV:3	*BOP1*	p.Val724Ile	8:144262235-C	[C*, T]	99	[20, 26]	missense_variant	0.00260000	22.6	4.704198
IV:5	*BOP1*	p.Val724Ile	8:144262235-C	[C*, T]	99	[22, 21]	missense_variant	0.00260000	22.6	4.704198
IV:3	*RNASE12*	p.Glu17Gly	14:20590674-T	[T*, C]	99	[16, 20]	missense_variant	0.00040000	24.6	7.158562
IV:5	*RNASE12*	p.Glu17Gly	14:20590674-T	[T*, C]	99	[19, 19]	missense_variant	0.00040000	24.6	7.158562
IV:3	*SPR*	p.Arg177Cys	2:72888538-C	[C*, T]	99	[12, 23]	missense_variant	0.00000000	34	8.467556
IV:5	*SPR*	p.Arg177Cys	2:72888538-C	[C*, T]	99	[12, 22]	missense_variant	0.00000000	34	8.467556
IV:3	*PHYHIPL*	p.Arg10Ser	10:59234305-G	[G*, T]	99	[10, 12]	missense_variant&splice_region_variant	0.00006172	10.27	8.774352
IV:5	*PHYHIPL*	p.Arg10Ser	10:59234305-G	[G*, T]	99	[13, 9]	missense_variant&splice_region_variant	0.00006172	10.27	8.774352
IV:3	*BAG2*	p.Val146Gly	6:57183991-T	[T*, G]	99	[15, 15]	missense_variant	0.00290000	13.75	9.106714
IV:5	*BAG2*	p.Val146Gly	6:57183991-T	[T*, G]	99	[12, 19]	missense_variant	0.00290000	13.75	9.106714
IV:3	*GNG8*	p.Val68Ile	19:46634087-C	[C*, T]	99	[22, 15]	missense_variant	0.00300000	14.63	11.213376
IV:5	*GNG8*	p.Val68Ile	19:46634087-C	[C*, T]	99	[17, 17]	missense_variant	0.00300000	14.63	11.213376
IV:3	*FAM57A*	p.Arg111Gln	17:737971-G	[G*, A]	99	[23, 15]	missense_variant	0.00070000	12.92	15.738610
IV:5	*FAM57A*	p.Arg111Gln	17:737971-G	[G*, A]	99	[7, 20]	missense_variant	0.00070000	12.92	15.738610
IV:3	*CARD18*	p.Glu40Lys	11:105138851-C	[C*, T]	99	[14, 19]	missense_variant	0.00340000	19.66	15.799969
IV:5	*CARD18*	p.Glu40Lys	11:105138851-C	[C*, T]	99	[22, 13]	missense_variant	0.00340000	19.66	15.799969
IV:3	*TDRP*	p.Lys197Gln	8:491580-T	[T*, G]	99	[21, 15]	missense_variant	0.00410000	22.7	16.756149
IV:5	*TDRP*	p.Lys197Gln	8:491580-T	[T*, G]	99	[10, 22]	missense_variant	0.00410000	22.7	16.756149
IV:3	*NTNG1*	p.Asn433Tyr	1:107436706-A	[A*, T]	99	[13, 19]	missense_variant	0.00020000	23.5	16.771488
IV:5	*NTNG1*	p.Asn433Tyr	1:107436706-A	[A*, T]	99	[17, 11]	missense_variant	0.00020000	23.5	16.771488
IV:3	*TM2D2*	p.Tyr145Cys	8:38991543-T	[C, C]	99	[0, 36]	missense_variant&splice_region_variant	0.00000000	25.0	19.409930

Case	Gene	Variant	Locus and Reference Allele	Allele 1 and Allele 2	GQ	AD	Impact	AF	CADD	GDI
IV:5	*TM2D2*	p.Tyr145Cys	8:38991543-T	[T*, C]	99	[21, 20]	missense_variant&splice_region_variant	0.00000000	25.0	19.409930
IV:3	*SIX3*	p.Ser88Leu	2:44942367-C	[C*, T]	99	[8, 12]	missense_variant	0.00330000	24.1	23.428951
IV:5	*SIX3*	p.Ser88Leu	2:44942367-C	[C*, T]	99	[11, 18]	missense_variant	0.00330000	24.1	23.428951
IV:3	*PDK1*	p.Ala386Ser	2:172592974-G	[G*, T]	99	[14, 9]	missense_variant	0.00030000	33	27.309915
IV:5	*PDK1*	p.Ala386Ser	2:172592974-G	[G*, T]	99	[10, 16]	missense_variant	0.00030000	33	27.309915
IV:3	*CIB2*	p.Arg155Gln	15:78105688-C	[C*, T]	99	[22, 20]	missense_variant	0.00430000	4.282	29.846091
IV:5	*CIB2*	p.Arg155Gln	15:78105688-C	[C*, T]	99	[17, 20]	missense_variant	0.00430000	4.282	29.846091
IV:3	*AP1B1*	p.Ala446Thr	22:29349319-C	[C*, T]	99	[15, 28]	missense_variant	0.00180000	34	31.579486
IV:5	*AP1B1*	p.Ala446Thr	22:29349319-C	[C*, T]	99	[16, 16]	missense_variant	0.00180000	34	31.579486
IV:3	*L3MBTL3*	p.Asn463His	6:130086194-A	[A*, C]	99	[20, 16]	missense_variant	0.00000000	26.7	32.269776
IV:5	*L3MBTL3*	p.Asn463His	6:130086194-A	[A*, C]	99	[11, 20]	missense_variant	0.00000000	26.7	32.269776
IV:3	*SAMD4B*	p.Pro419Leu	19:39377636-C	[C*, T]	99	[22, 18]	missense_variant	0.00180000	8.088	32.494759
IV:5	*SAMD4B*	p.Pro419Leu	19:39377636-C	[C*, T]	99	[17, 18]	missense_variant	0.00180000	8.088	32.494759
IV:3	*LRRC73*	p.Thr83Ile	6:43509538-G	[G*, A]	99	[21, 19]	missense_variant	0.00030000	21.6	35.097408
IV:5	*LRRC73*	p.Thr83Ile	6:43509538-G	[G*, A]	99	[32, 23]	missense_variant	0.00030000	21.6	35.097408
IV:3	*SCMH1*	p.Ser19Thr	1:41152620-A	[A*, T]	99	[11, 10]	missense_variant	0.00260000	9.880	36.554686
IV:5	*SCMH1*	p.Ser19Thr	1:41152620-A	[A*, T]	99	[18, 17]	missense_variant	0.00260000	9.880	36.554686
IV:3	*DGKD*	p.Val1038Ile	2:233462661-G	[G*, A]	99	[14, 18]	missense_variant	0.00080000	23.1	37.142711
IV:5	*DGKD*	p.Val1038Ile	2:233462661-G	[G*, A]	99	[16, 24]	missense_variant	0.00080000	23.1	37.142711
IV:3	*CDK10*	p.Leu21Val	16:89687600-C	[C*, G]	99	[31, 27]	missense_variant&NMD_transcript_variant	0.00030000	.	37.316562
IV:5	*CDK10*	p.Leu21Val	16:89687600-C	[C*, G]	99	[14, 19]	missense_variant&NMD_transcript_variant	0.00030000	.	37.316562
IV:3	*CDK7*	p.Leu277Ser	5:69273007-T	[T*, C]	99	[14, 16]	missense_variant	0.00000000	15.89	37.976172

Case	Gene	Variant	Locus and Reference Allele	Allele 1 and Allele 2	GQ	AD	Impact	AF	CADD	GDI
IV:5	*CDK7*	p.Leu277Ser	5:69273007-T	[T*, C]	99	[11, 15]	missense_variant	0.00000000	15.89	37.976172
IV:3	*CDC25A*	p.Glu492Lys	3:48159046-C	[C*, T]	99	[24, 20]	missense_variant	0.00030000	27.4	38.594877
IV:5	*CDC25A*	p.Pro20Ser	3:48187890-G	[G*, A]	99	[29, 34]	missense_variant	0.00080000	0.154	38.594877
IV:3	*SP100*	p.Arg838Cys	2:230542000-C	[C*, T]	99	[18, 15]	missense_variant	0.00100000	29.2	40.302705
IV:5	*SP100*	p.Glu790Lys	2:230515368-G	[G*, A]	99	[21, 20]	missense_variant	0.00200000	10.22	40.302705
IV:5	*SP100*	p.Arg838Cys	2:230542000-C	[C*, T]	99	[21, 23]	missense_variant	0.00100000	29.2	40.302705
IV:3	*DDX10*	p.Ile240Thr	11:108679431-T	[T*, C]	99	[11, 13]	missense_variant	0.00020000	25.7	40.824257
IV:5	*DDX10*	p.Ile240Thr	11:108679431-T	[T*, C]	99	[14, 20]	missense_variant	0.00020000	25.7	40.824257
IV:3	*EIF4ENIF1*	p.Met610Thr	22:31448172-A	[A*, G]	99	[19, 24]	missense_variant	0.00040000	17.23	41.463415
IV:5	*EIF4ENIF1*	p.Met610Thr	22:31448172-A	[A*, G]	99	[17, 19]	missense_variant	0.00040000	17.23	41.463415
IV:3	*ACOT6*	p.His52Arg	14:73619370-A	[A*, G]	99	[20, 13]	missense_variant	0.00340000	5.237	42.567879
IV:5	*ACOT6*	p.His52Arg	14:73619370-A	[A*, G]	99	[18, 16]	missense_variant	0.00340000	5.237	42.567879
IV:3	*PLAU*	p.Arg201Leu	10:73913680-G	[G*, T]	99	[16, 22]	missense_variant	0.00220000	23.8	44.142762
IV:5	*PLAU*	p.Arg201Leu	10:73913680-G	[T, T]	99	[0, 48]	missense_variant	0.00220000	23.8	44.142762
IV:3	*PSMB11*	p.Thr14Ser	14:23042265-A	[A*, T]	99	[24, 16]	missense_variant	0.00030000	0.048	44.613182
IV:5	*PSMB11*	p.Thr14Ser	14:23042265-A	[A*, T]	99	[16, 18]	missense_variant	0.00030000	0.048	44.613182
IV:3	*FAM126A*	p.Tyr27His	7:22984018-A	[A*, G]	99	[16, 12]	missense_variant	0.00000000	27.6	46.121593
IV:5	*FAM126A*	p.Tyr27His	7:22984018-A	[A*, G]	99	[17, 18]	missense_variant	0.00000000	27.6	46.121593
IV:3	*SNAI3*	p.Arg268Gln	16:88678524-C	[C*, T]	99	[23, 19]	missense_variant	0.00150000	1.360	46.464182
IV:5	*SNAI3*	p.Arg268Gln	16:88678524-C	[C*, T]	99	[30, 25]	missense_variant	0.00150000	1.360	46.464182
IV:3	*ZC3H18*	p.Pro885Leu	16:88630572-C	[C*, T]	99	[15, 27]	missense_variant	0.00260000	7.829	47.164698
IV:5	*ZC3H18*	p.Pro885Leu	16:88630572-C	[C*, T]	99	[25, 20]	missense_variant	0.00260000	7.829	47.164698

Case	Gene	Variant	Locus and Reference Allele	Allele 1 and Allele 2	GQ	AD	Impact	AF	CADD	GDI
IV:3	*MFHAS1*	p.Asn856Asp	8:8890493-T	[T*, C]	99	[13, 21]	missense_variant	0.00070000	13.37	47.808969
IV:5	*MFHAS1*	p.Asn856Asp	8:8890493-T	[T*, C]	99	[16, 22]	missense_variant	0.00070000	13.37	47.808969
IV:3	*NRP2*	p.Leu441Phe	2:205743232-C	[C*, T]	99	[18, 8]	missense_variant	0.00280000	25.1	47.947027
IV:5	*NRP2*	p.Leu441Phe	2:205743232-C	[C*, T]	99	[19, 12]	missense_variant	0.00280000	25.1	47.947027
IV:3	*ARHGAP5*	p.Ile1502Met	14:32154945-A	[A*, G]	99	[21, 12]	missense_variant	0.00200000	22.8	48.662883
IV:5	*ARHGAP5*	p.Pro1466Leu	14:32154836-C	[C*, T]	99	[12, 12]	missense_variant	0.00030000	24.5	48.662883
IV:5	*ARHGAP5*	p.Ile1502Met	14:32154945-A	[A*, G]	99	[13, 12]	missense_variant	0.00200000	22.8	48.662883
IV:3	*USP32*	p.Leu965Phe	17:60208091-G	[G*, A]	99	[16, 8]	missense_variant	0.00030000	28.3	49.266247
IV:5	*USP32*	p.Leu965Phe	17:60208091-G	[G*, A]	99	[14, 11]	missense_variant	0.00030000	28.3	49.266247
IV:3	*PTPRE*	p.Gly416Ser	10:128070403-G	[G*, A]	99	[15, 20]	missense_variant	0.00060000	5.582	49.312267
IV:5	*PTPRE*	p.Gly416Ser	10:128070403-G	[G*, A]	99	[18, 17]	missense_variant	0.00060000	5.582	49.312267
IV:3	*RABEP1*	p.Asp70Asn	17:5331993-G	[G*, A]	99	[8, 13]	missense_variant	0.00100000	25.1	49.322493
IV:5	*RABEP1*	p.Asp70Asn	17:5331993-G	[G*, A]	99	[24, 15]	missense_variant	0.00100000	25.1	49.322493
IV:3	*ZBTB11*	p.Asn428Ser	3:101665304-T	[T*, C]	99	[16, 20]	missense_variant	0.00100000	0.001	50.943396
IV:5	*ZBTB11*	p.Asn428Ser	3:101665304-T	[T*, C]	99	[16, 14]	missense_variant	0.00100000	0.001	50.943396
IV:3	*MBOAT4*	p.Ser278Arg	8:30132417-G	[T, T]	93	[0, 31]	missense_variant	0.00060000	1.966	51.812650
IV:5	*MBOAT4*	p.Ser278Arg	8:30132417-G	[G*, T]	99	[16, 23]	missense_variant	0.00060000	1.966	51.812650
IV:3	*CDH4*	p.Val640Ile	20:61928336-G	[G*, A]	99	[22, 19]	missense_variant	0.00120000	10.54	53.157437
IV:5	*CDH4*	p.Val640Ile	20:61928336-G	[G*, A]	99	[21, 23]	missense_variant	0.00120000	10.54	53.157437
IV:3	*LSP1*	p.Gly138Arg	11:1866886-G	[G*, A]	99	[28, 28]	missense_variant	0.00310000	3.566	54.169862
IV:5	*LSP1*	p.Gly138Arg	11:1866886-G	[G*, A]	99	[26, 24]	missense_variant	0.00310000	3.566	54.169862
IV:3	*TRIM25*	p.Lys313Glu	17:56901569-T	[T*, C]	99	[15, 12]	missense_variant	0.00170000	23.2	55.739633

Case	Gene	Variant	Locus and Reference Allele	Allele 1 and Allele 2	GQ	AD	Impact	AF	CADD	GDI
IV:5	*TRIM25*	p.Lys313Glu	17:56901569-T	[T*, C]	99	[23, 16]	missense_variant	0.00170000	23.2	55.739633
IV:3	*BTD*	p.Tyr454Cys	3:15645217-A	[A*, G]	99	[11, 16]	missense_variant	0.00210000	24.4	56.342997
IV:5	*BTD*	p.Tyr454Cys	3:15645217-A	[A*, G]	99	[20, 15]	missense_variant	0.00210000	24.4	56.342997
IV:3	*USP20*	p.Tyr663Cys	9:129874895-A	[A*, G]	99	[20, 19]	missense_variant	0.00070000	23.6	57.621312
IV:5	*USP20*	p.Tyr663Cys	9:129874895-A	[A*, G]	99	[25, 13]	missense_variant	0.00070000	23.6	57.621312
IV:3	*ZFYVE16*	p.Gln26Pro	5:80436762-A	[A*, C]	99	[13, 18]	missense_variant	0.00260000	21.3	58.991665
IV:5	*ZFYVE16*	p.Gln26Pro	5:80436762-A	[A*, C]	99	[8, 11]	missense_variant	0.00260000	21.3	58.991665
IV:3	*MTDH*	p.Ser529Cys	8:97722943-C	[C*, G]	99	[14, 17]	missense_variant	0.00230000	28.1	59.073478
IV:5	*MTDH*	p.Ser529Cys	8:97722943-C	[C*, G]	99	[19, 20]	missense_variant	0.00230000	28.1	59.073478
IV:3	*SRCIN1*	p.Arg816His	17:38552480-C	[C*, T]	99	[22, 19]	missense_variant	0.00200000	33	60.990950
IV:5	*SRCIN1*	p.Arg816His	17:38552480-C	[C*, T]	99	[25, 24]	missense_variant	0.00200000	33	60.990950
IV:3	*SYNJ2*	p.Glu66Lys	6:158017272-G	[G*, A]	99	[22, 16]	missense_variant	0.00010000	24.9	61.011403
IV:5	*SYNJ2*	p.Glu66Lys	6:158017272-G	[G*, A]	99	[13, 19]	missense_variant	0.00010000	24.9	61.011403
IV:3	*SYT5*	p.Gly38Arg	19:55179094-C	[C*, T]	99	[12, 20]	missense_variant	0.00290000	14.20	62.202792
IV:5	*SYT5*	p.Gly38Arg	19:55179094-C	[C*, T]	99	[14, 23]	missense_variant	0.00290000	14.20	62.202792
IV:3	*IL17REL*	p.Arg320His	22:49997335-C	[C*, T]	99	[22, 19]	missense_variant	0.00280000	6.559	62.274377
IV:5	*IL17REL*	p.Arg320His	22:49997335-C	[C*, T]	99	[18, 21]	missense_variant	0.00280000	6.559	62.274377
IV:3	*ETV7*	p.Pro296Ser	6:36366897-G	[G*, A]	99	[17, 12]	missense_variant	0.00200000	26.9	63.235670
IV:5	*ETV7*	p.Pro296Ser	6:36366897-G	[G*, A]	99	[14, 19]	missense_variant	0.00200000	26.9	63.235670
IV:3	*BRPF3*	p.Pro834Leu	6:36213898-C	[C*, T]	99	[17, 13]	missense_variant	0.00130000	18.32	63.685637
IV:5	*BRPF3*	p.Pro834Leu	6:36213898-C	[C*, T]	99	[16, 23]	missense_variant	0.00130000	18.32	63.685637
IV:3	*TBC1D9B*	p.Glu1083del	5:179863949-GTCC	[GTCC*, G]	99	[18, 14]	inframe_deletion	0.00140000	.	65.066217

Case	Gene	Variant	Locus and Reference Allele	Allele 1 and Allele 2	GQ	AD	Impact	AF	CADD	GDI
IV:5	*TBC1D9B*	p.Glu1083del	5:179863949-GTCC	[GTCC*, G]	99	[22, 15]	inframe_deletion	0.00140000	.	65.066217
IV:3	*BIRC6*	p.Met4560Ile	2:32597818-G	[G*, A]	91	[6, 16]	missense_variant	0.00470000	23.0	65.756507
IV:5	*BIRC6*	p.Met4560Ile	2:32597818-G	[G*, A]	99	[15, 15]	missense_variant	0.00470000	23.0	65.756507
IV:3	*LRIG2*	p.Val929Ile	1:113119337-G	[G*, A]	99	[11, 14]	missense_variant	0.00200000	17.90	66.656440
IV:5	*LRIG2*	p.Val929Ile	1:113119337-G	[A, A]	74	[0, 25]	missense_variant	0.00200000	17.90	66.656440
IV:3	*USP14*	p.Pro350Ser	18:204576-C	[C*, T]	99	[15, 13]	missense_variant	0.00140000	26.7	67.730224
IV:5	*USP14*	p.Pro350Ser	18:204576-C	[C*, T]	99	[20, 29]	missense_variant	0.00140000	26.7	67.730224
IV:3	*SHROOM1*	p.Ser408Tyr	5:132824633-G	[G*, T]	99	[16, 17]	missense_variant	0.00070000	11.15	68.144398
IV:3	*SHROOM1*	p.Ser408Thr	5:132824634-A	[A*, T]	99	[16, 17]	missense_variant	0.00070000	7.570	68.144398
IV:5	*SHROOM1*	p.Ser408Tyr	5:132824633-G	[G*, T]	99	[15, 18]	missense_variant	0.00070000	11.15	68.144398
IV:5	*SHROOM1*	p.Ser408Thr	5:132824634-A	[A*, T]	99	[15, 17]	missense_variant	0.00070000	7.570	68.144398
IV:3	*GIPC1*	p.Arg159His	19:14480484-C	[C*, T]	99	[25, 21]	missense_variant&splice_region_variant	0.00050000	33	68.737536
IV:5	*GIPC1*	p.Arg159His	19:14480484-C	[C*, T]	99	[20, 19]	missense_variant&splice_region_variant	0.00050000	33	68.737536
IV:3	*ATP6V0E2*	p.Asp171Tyr	7:149879546-G	[G*, T]	99	[22, 17]	missense_variant	0.00170000	22.6	69.192616
IV:5	*ATP6V0E2*	p.Asp171Tyr	7:149879546-G	[T, T]	99	[0, 38]	missense_variant	0.00170000	22.6	69.192616
IV:3	*GEMIN5*	p.Arg66Lys	5:154937155-C	[C*, T]	99	[21, 12]	missense_variant	0.00200000	15.09	69.908473
IV:5	*GEMIN5*	p.Arg66Lys	5:154937155-C	[C*, T]	99	[16, 21]	missense_variant	0.00200000	15.09	69.908473
IV:3	*FXYD5*	p.Thr105Met	19:35164177-C	[C*, T]	99	[17, 25]	missense_variant	0.00100000	24.8	70.307307
IV:5	*FXYD5*	p.Thr105Met	19:35164177-C	[C*, T]	99	[16, 12]	missense_variant	0.00100000	24.8	70.307307
IV:3	*PER3*	p.Thr1120Ile	1:7835906-C	[T, T]	81	[0, 27]	missense_variant	0.00460000	11.93	70.736821
IV:5	*PER3*	p.Thr1120Ile	1:7835906-C	[C*, T]	99	[23, 17]	missense_variant	0.00460000	11.93	70.736821
IV:3	*TTBK2*	p.Asp1399Tyr	15:42752266-C	[A, A]	93	[0, 31]	missense_variant	0.00430000	29.2	71.723680

Case	Gene	Variant	Locus and Reference Allele	Allele 1 and Allele 2	GQ	AD	Impact	AF	CADD	GDI
IV:5	*TTBK2*	p.Asp1399Tyr	15:42752266-C	[C*, A]	99	[18, 19]	missense_variant	0.00430000	29.2	71.723680
IV:3	*RASAL3*	p.Ala233Val	19:15458620-G	[G*, A]	99	[18, 17]	missense_variant	0.00290000	16.09	72.173646
IV:5	*RASAL3*	p.Ala233Val	19:15458620-G	[G*, A]	99	[19, 13]	missense_variant	0.00290000	16.09	72.173646
IV:3	*CAST*	p.Ile120Val	5:96730837-A	[A*, G]	99	[19, 16]	missense_variant	0.00030000	0.001	72.383290
IV:5	*CAST*	p.Ile120Val	5:96730837-A	[A*, G]	99	[19, 22]	missense_variant	0.00030000	0.001	72.383290
IV:3	*FLT4*	p.Arg1320Trp	5:180603326-G	[G*, A]	99	[10, 21]	missense_variant	0.00170000	24.9	72.833257
IV:5	*FLT4*	p.Arg1320Trp	5:180603326-G	[G*, A]	99	[27, 25]	missense_variant	0.00170000	24.9	72.833257
IV:3	*GLTPD2*	p.Leu105Pro	17:4789653-T	[C, C]	99	[0, 44]	missense_variant	0.00360000	24.8	72.869049
IV:5	*GLTPD2*	p.Leu105Pro	17:4789653-T	[T*, C]	99	[15, 24]	missense_variant	0.00360000	24.8	72.869049
IV:3	*PRX*	p.Gly1286Ser	19:40394496-C	[C*, T]	99	[24, 18]	missense_variant	0.00330000	21.9	73.053127
IV:5	*PRX*	p.Gly1286Ser	19:40394496-C	[C*, T]	99	[19, 23]	missense_variant	0.00330000	21.9	73.053127
II:3	*LIPE*	p.Ala418Thr	19:42410474-C	[C*, T]	99	[30, 23]	missense_variant	0.00150000	22.7	73.206525
IV:5	*LIPE*	p.Ala418Thr	19:42410474-C	[C*, T]	99	[17, 20]	missense_variant	0.00150000	22.7	73.206525
IV:3	*UNC45A*	p.Ala692Val	15:90950155-C	[C*, T]	99	[16, 15]	missense_variant & splice_region_variant	0.00130000	21.1	73.743417
IV:5	*UNC45A*	p.Ala692Val	15:90950155-C	[C*, T]	99	[24, 24]	missense_variant &splice_region_variant	0.00130000	21.1	73.743417
IV:3	*CC2D2A*	p.Arg109Gln	4:15481275-G	[G*, A]	99	[15, 16]	missense_variant	0.00030000	1.024	73.891701
IV:5	*CC2D2A*	p.Arg109Gln	4:15481275-G	[G*, A]	99	[13, 11]	missense_variant	0.00030000	1.024	73.891701
IV:3	*HFM1*	p.Lys1193Glu	1:91276639-T	[T*, C]	99	[11, 12]	missense_variant	0.00350000	29.4	73.968400
IV:5	*HFM1*	p.Lys1193Glu	1:91276639-T	[T*, C]	99	[19, 15]	missense_variant	0.00350000	29.4	73.968400

GQ, genotype quality; AD, allelic depth; AF, allele frequency; NMD, nonsense mediated decay; CADD, Combined Annotation Dependent Depletion; GDI, Gene Damage Index

Supplementary Table 6. Autosomal recessive model: missense and LOF from genome sequencing data

Case	Gene	Variant	Locus and Reference Allele	Allele 1 and Allele 2	GQ	AD	Impact	AF	CADD	GDI
IV:5	*NME4*	c.517-1G>A	16:401946-G	[A, A]	99	[0, 42]	splice_acceptor_variant & NMD_transcript_variant	0.0003	.	13.23823
IV:3	*TM2D2*	p.Tyr145Cys	8:38991543-T	[C, C]	99	[0, 36]	missense_variant & splice_region_variant	0.0000	25.0	19.40993
IV:5	*C16orf58*	p.Arg293Trp	16:31493684-G	[A, A]	99	[0, 40]	missense_variant	0.0049	23.0	40.80380
IV:5	*PLAU*	p.Arg201Leu	10:73913680-G	[T, T]	99	[0, 48]	missense_variant	0.0022	23.8	44.14276
IV:3	*MBOAT4*	p.Ser278Arg	8:30132417-G	[T, T]	93	[0, 31]	missense_variant	0.0006	1.966	51.81265
IV:5	*ZNF277*	p.Ser272Leu	7:112336117-C	[T, T]	99	[0, 43]	missense_variant	0.0004	26.4	60.91425
IV:5	*ATP6V0E 2*	p.Asp171Tyr	7:149879546-G	[T, T]	99	[0, 38]	missense_variant	0.0017	22.6	69.19262
IV:3	*PER3*	p.Thr1120Ile	1:7835906-C	[T, T]	81	[0, 27]	missense_variant	0.0046	11.93	70.73682
IV:3	*TTBK2*	p.Asp1399Tyr	15:42752266-C	[A, A]	93	[0, 31]	missense_variant	0.0043	29.2	71.72368
IV:3	*GLTPD2*	p.Leu105Pro	17:4789653-T	[C, C]	99	[0, 44]	missense_variant	0.0036	24.8	72.86905

GQ, genotype quality; AD, allelic depth; AF, >allele frequency; NMD, nonsense mediated decay; CADD, Combined Annotation Dependent Depletion; GDI, Gene Damage Index
Note: USP53 was excluded because GDI > 75th percentile (threshold)

Supplementary Table 7. Antibodies used & concentrations.

Antibody (species, conjugate)	Commercial concentration	Concentration applied	Source
Rabbit α-human USP53 (PrEST)3	0.2 mg/ml	1-4 µg/ml	Sigma life science: Product number: HPA035844
Mouse α-human USP53	0.05 mg/ml	5-20 ug/ml	Novus H00054532-B01P
α-Rabbit GRIP2	0.48 mg/ml	1-4 µg/ml	Invitrogen Thermo Fisher scientific Catalogue: PA5-48489
α -Mouse GluA2	1mg/ml	1-10 µg/ml	UC Davis/NIH NeuroMab Facility 75002020; 472-1JU-17
α -Rabbit KLH	1mg/ml	1-10 µg/ml	A150-104A BETHYL, Hamburg, Germany
[Alexa Fluor 488]-conjugated goat α-rabbit IgG	1 mg/ml	2 µg/ml	ThermoFisher Scientific, Waltham, MA # A32731
[Alexa Fluor Plus 594]-conjugated goat α-mouse IgG	1 mg/ml	2 µg/ml	ThermoFisher Scientific, Waltham, MA # A32742
Peroxidase conjugated goat α-rabbit IgG (H+L)	10 µg/ml	0.1 µg/ml	Invitrogen/Thermo Fisher Scientific # 32460
Peroxidase conjugated goat α-mouse IgG (H+L)	100 µg/ml	0.125 µg/ml	Cell Signaling Technology (Danvers, MA) # 7076
Peroxidase conjugated goat α-mouse IgG (L)	5.4 µg /ml	0.05-0.2 µg /ml	Cell Signaling Technology #91196

α-, anti-; HRP, horse radish peroxidase conjugated; KLH, keyhole limpet hemocyanin; H, heavy chain; L, light chain specific

Supplementary Table 8. Antibodies used in immunohistochemistry and western blots.

Antibody	Primary Antibody	Source	Description	Characterization
Primary	α-USP53	Sigma Life Science: HPA0035844	Prestige validated, affinity purified, polyclonal rabbit α-human PrEST USP53 (tail)) Lot R42677	Kamierczak et al., 2015, Fig 8; Kanwal et al. D-F
Primary	α-USP53	Novus-Biologicals H00054532-B01P	mouse α-human USP53; synthetic peptide (1-188); catalytic domain)	Manufacturer
Primary	α-USP53/preabsorbed	Sigma HPA0035844 + 10xAg	Ten-fold excess APrEST78787 added to α-USP53	Kanwal et al., Fig. 5M
Primary	α-GRIP2	Invitrogen PA5-48489 Lot XD356890	rabbit polyclonal α-human GRIP2—the antigen was a KLH-conjugated, synthetic peptide (620-649) Protein A affinity.	Manufacturer; Kanwal et al., Fig. 5
Primary	α-KLH	Bethyl A150-104A-7	rabbit polyclonal α-keyhole limpet hemocyanin affinity purified by Bethyl Labs A150-104A-7	Kanwal et al., Fig. 5N
Primary	α-GRIA2	Antibodies Inc 75-002-020	mouse α-GRIA2; Clone L31/32	KO validated; Deposited by Trimmer,J.S.-UC Davis
Secondary	Alexa Fluor 488 goat α-rabbit IgG (H/L)	Thermo Fisher A32731	cross-absorbed	Manufacturer
Secondary	Alexa Fluor 594 goat α-mouse IgG (H/L)	Thermo Fisher # A32742	cross-absorbed	Manufacturer
Secondary	HRP goat α-rabbit IgG (H/L)	Cell Signaling #7074	cross-absorbed	Manufacturer
Secondary	HRP horse α-mouse IgG (H/L)	Cell Signaling #7076	cross-absorbed	Manufacturer
Secondary	HRP goat α-mouse IgG(L)	Cell Signaling #91196	cross-absorbed	Manufacturer

α-, anti-; HRP, horse radish peroxidase conjugated; KLH, keyhole limpet hemocyanin; H, heavy chain; L, light chain

SUPPLEMENTARY MATERIALS AND METHODS

Human Participants

The study was approved by the institutional review board at the School of Biological Sciences, University of the Punjab, IRB # 00005281. Written informed consent was obtained from the participants.

A family, PSY01 (Figure 1A), was identified from the outpatient department at a local hospital. Two siblings carried the diagnosis of severe schizophrenia. Hearing was assessed normal by observation (see below), since the subjects refused audiometry. The family denied history of jaundice during proband's infancy or childhood (see below). Standard rating scales and structured interviews were used. Interviews lasted approximately 3.5 hours. Additional details about participant recruitment, assessment, interviews, and other special circumstances have been published elsewhere (1).

Genetic analyses

DNA was extracted using sucrose lysis followed by salting out (2). Quality of the genomic DNA was assessed by electrophoresis on a 1% agarose gel. Samples of both affected individuals and father (Figure 1A) were analyzed by exome and whole genome sequencing which was performed commercially (Macrogen, Seoul, South Korea). All genotypes in the pedigree were confirmed with Sanger sequencing.

Whole-exome sequencing (WES)

Whole-exome sequencing followed exome capture (Agilent SureSelect XT Human All Exon v5) and bridge amplification using the Illumina HiSeq 2500 with paired-end reads at 100X depth, and the data were mapped to GRCh37/hg19 assembly. Typical

sequencing output parameters are displayed (Supplementary Table 5). The variant call files (vcf) were analyzed using wANNOVAR (https://wannovar.wglab.org/) and Franklin software (https://franklin.genoox.com/clinical-db/home). Variants were filtered so that those with an allele frequency of 0.01 or higher in public databases (gnomAD, 1000 Genomes, ESP 6500, TOPMeD, Bravo, GenomeAsia, jMorp, Iranome, GME Variome) were removed. Prioritized variants from the exome data were homozygous in both affected individuals and heterozygous in the father. All exonic variants and those located up to ±10 nucleotides in the intronic regions relative to the exons were selected. In addition, homozygosity mapping was also performed on exome data using AgileVCFMapper to identify the runs of homozygosity shared by both affected individuals but heterozygous in the parent. All intronic variants located in these regions were also examined.

The wANNOVAR files or those from Franklin included predicted pathogenic scores for these variants from many software including Polyphen-2, Mutation Taster, REVEL, SIFT, and the pathogenicity CADD scores indicating the probable impact of variation on the function of the encoded protein. The conservation of selected amino acid residues affected by variants was checked across vertebrate species. For this purpose, multiple alignments were carried out using ClustalO on the protein sequences obtained from UniProt.

Prioritized variants from the hg19 exome data (Supplementary Table 3) were checked in the family for segregation by Sanger sequencing (Supplementary Figure 4) in DNA samples from all participants. Control frequencies in the

population were assessed by competitive ARMS primers PCR (3) in 400 ethnically matched, indigenous individuals.

Whole genome sequencing

Whole genome sequencing (WGS) followed the Illumina (San Diego, CA) pipeline with fragmentation of 100 ng of genomic DNA using adaptive focused acoustic technology (AFA; Covaris, Woburn, MA). TrueSeq was used for creation of the library. The fragmented DNA was end-repaired to create 5´-phosphorylated, blunt-ended, double-stranded DNA molecules. Following end-repair, DNA was selected for size with a magnetic bead-based method. These DNA fragments went through the addition of a single 'A' base, and ligation of indexing adapters. The purified libraries were quantified using qPCR according to the qPCR Quantification Protocol Guide (KAPA Library Quantification kits for Illumina Sequencing platforms) and qualified using the Agilent Technologies 2200 TapeStation (Agilent, Santa Clara, CA). WGS analysis followed the GATK pipeline with variant selection detailed further below.

Whole Genome Sequencing Data Analysis

Pre-processing

The general GATK workflow for discovery of germline variants maps the raw reads to the GRCh38/hg38 reference genome producing an aligned file (sam/bam) sorted by coordinate. Duplicate reads were marked to mitigate biases from procedures such as PCR amplification. The base quality scores, critical for variant calling algorithms, require recalibration based on covariate measurements of calls in the dataset and in each sample. This recalibration addresses biases from sample preparation or

While our primary search was limited to variants inherited in an autosomal recessive fashion, we considered a total of five genetic models.

1) *Autosomal Dominant or Multiple Hits: Genes with two high impact (i.e., LoF) variants;* allelic depth (AD) >20; genotype quality (GQ) >50; and gene damage index (GDI) <75 percentile. 2) *Variants of Interest (VOI) or previously reported genes*: one high impact and one moderate impact (e.g., missense); AF <0.005; no quality filters. 3) *Autosomal Recessive*: one high impact and one moderate impact variant; AD >20; GQ >50; AF<0.005; and GDI <75 percentile. 4) *X-linked*: *Autosomal Recessive*: one high impact and one moderate impact homozygous X-linked variant; AD >20; GQ >50; AF<0.005; and GDI <75 percentile. 5) *Compound Heterozygote.* One or more high impact variants and one or more moderate impact variants in the same sample on separate haplotypes as determined by IGV.

Each variant was then manually reviewed using IGV to confirm not an artifact or other cause for concern. Sporadic variant calls littering the area around the SNV were considered suspect and were checked against an existing database (gnomAD, BRAVO). Variants were reported if the number of *good* calls matched the corresponding requirements of their source (i.e., genetic model).

CV discovery

Model free analysis

CNVs were called from the vcf output from smoove using the aligned reads (bam files) after pre-processing as above. CNVs were filtered using duphold to select

deletions (duplications) with <0.7-fold change (>1.3-fold change, respectively) for variant depth relative to flanking regions or for genomic regions with similar GC content. AnnotSV was used for annotation of the filtered CNVs and for assignment to a category of pathogenicity: benign, likely benign, variant of unknown significance, likely pathogenic, and pathogenic. Pathogenic and likely pathogenic CNVs as well as any CNV around the genomic coordinates of the VOI (*USP53*) were examined and visualized with samplot gene. Of note, no CNV in the VOI region was called for any of the samples. Five CNV regions were called as likely pathogenic or pathogenic by AnnotSV: a) two pathogenic CNVs occurred in chromosome 4 but were present in all samples: a 304 bp deletion and a 117 kb deletion (chr4: 68508074-68625397); b) chr19: 42813046-43241094 (pathogenic) was found in only one of the affected daughters; c) two potentially pathogenic overlapping regions on chr20:29125607-30424527 and chr20: 29294818-30482698 were blacklisted (and excluded by Genvisis) and not examined further. A deletion (chr4:118712587-119795518; USP53 region +/- 500kb) was not called in the CNV set but was visualized.

Pedigree-based model: Rare & pathogenic.

Because the two daughters are affected and the father is not, the vcf output from smoove was filtered additionally using the slivar tool for paternal heterozygosity and homozygosity in both affected daughters. The analysis above (model free) found only one CNV present in father and both affected daughters.

Pedigree-based model: Rare & unknown pathogenicity.

The vcf filtered for paternal heterozygosity and homozygosity in affected daughters was searched with AnnotSV for deletions and duplications that were not annotated as

benign loss or benign gain, respectively. The outcome was a set of 39 CNV regions with unknown significance for pathogenicity. These CNVs were inspected using samplot. Five of the 39 CNVs were blacklisted (i.e., location in low complexity regions; incomplete reference genome assembly; bioinformatic misprocessing, or limitations inherent to cohort-specific private alleles--e.g., arising from genetic ancestries or sequencing kits) (64) and were not considered further: chr10:132914133-132914719; chr13:113136636-113136949; chr7:58189288-61967062; chr7:60039029-60527184; chr7:60527462-6053519.

Immunohistochemistry

All animal studies were performed under protocols approved by the IACUC. CB57Bl/6J mice (either sex, age ~ 3 months) were anesthetized (i.p. ketamine/xylazine), and the chest was opened to perfuse the left ventricle with cold phosphate buffered saline (1XPBS) containing 4% paraformaldehyde (PFA). After dissection, the brain was fixed in cold PBS/4%PFA for 24 hours followed by treatment for 24 h with PBS containing 2% PFA/15% sucrose. Subsequently , the brain was transferred to 30% sucrose in PBS for 24 hours. The whole brain was embedded with its ventral surface on a chuck using Tissue-Tek O.C.T. Compound (Sakura, Torrence, CA) and was frozen in the cryostat at -21 °C. Transverse frozen sections (including cerebellum and olfactory bulb) were ~ 30 µm thick and and were air-dried overnight and mounted unto positively charged glass slides then stored at -80 °C.

IF histochemistry was performed as follows. Slide sections were permeabilized in PBS with 0.1% Triton X-100 (PBST) at room temperature (RT) for 10 min and then blocked with 1% goat serum in PBS at RT for 30 min followed by drainage of excess

liquid. Sections were overlaid with 100 µl primary antibody and incubated overnight at 4 °C. The sections were washed thrice with 1XPBS, each for 5 min. They were incubated at RT after being overlaid with 100 µl of secondary antibody. The concentrations used for IF of both primary and secondary antibodies were 1-2 µg/ml.

Slides for anatomical identification were stained with crystal violet acetate (Nissl). Slides for IF were mounted with Fluoromount-G (Invitrogen, Waltham, MA) and glass coverslips. Sections were visualized with Evos FL Auto Fluoresence/Light microscope (ThermoFisher, Waltham , MA) with 4X (NA 0.13), 10X (NA 0.4), 20X (NA 0.8), and 40X (0.95) plan-apochromat objectives.

Co-immunoprecipitation (co-IP) & western blots (WB)

All procedures were done on ice and at 4° C. The dissected brain (~ 500 mg) was added to 0.5 ml cold Syn-PER™ Synaptic Protein Extraction Reagent (SSPER, Thermo Fisher (Waltham, MA). Homogenization was performed with 10 passes using a 1 ml Dounce tissue grinder (Wheaton, Millville, NJ) with a tight pestle. After centrifugation at 1,200 g for 10 min, the pellet (P1; i.e., debris containing nuclei, mitochondria, etc., and noted to contain minimal USP53) was discarded. The supernatant (S1) was centrifuged at 15,000 g for 20 min at 4° C. The cytosolic fraction (S2) was discarded, while the pellet (P2) containing synaptosomes was resuspended in 200 µl SSPER and 100 µl co-IP buffer [150 mM NaCl, 10 mm Tris-HCl pH 7.4, 1 mM EDTA, 1 mM EGTA, 1x HALT inhibitor cocktail (Thermo fisher, Waltham, MA), Triton X-100 1%, NP-40 0.5%].

The remaining procedures followed the company's protocol for extraction of immune complexes with 25 µl Protein A magnetic beads (Pierce) except the amount of primary antibody was 2.88 µg. The beads were resuspended in 30 µl 3X Laemmli buffer before separation on SDS-PAGE and subsequent transfer to a PVDF membrane.

After transfer of proteins, the membrane was treated with blocking buffer (5% BSA in 1X Tris Buffered Saline-Tween 20 [TBST]: 20 mM Tris base, 137 mM NaCl, 38 mM HCl, Tween 20 0.5%) for 60 min at room temperature with rocking. Subsequently, the membrane was incubated with the primary antibody in 10 ml 1X TBST for 60 minutes at room temperature followed by four subsequent washes with 1X TBST each for 5 min. To probe the blot, the secondary antibody [stabilized peroxidase conjugated goat anti-rabbit (H+L) HRP secondary antibody; Invitrogen/Thermo Fisher Scientific # 32460; stock 10 ug/ml; 1:1000 in TBST] was added for 60 min at room temperature with rocking. Subsequently, the membrane was washed four times with TBST by rocking for 5 min each. The bands were detected using chemiluminescence with SuperSignal™ West Dura (Thermo Fisher Scientific) and recorded using a Li-Cor system (Lincoln, Nebraska).

Antibodies for IF and IP

Primary antibodies included the following: 1) polyclonal rabbit anti-human PrEST USP53; Sigma HPA035844, Lot R32677 (St. Louis, MO); USP53 antibody did not cross-react to GRIP2 as confirmed with western blot (WB) ; 1 µg/ml PBST, 1% goat serum, 1% BSA. 2) rabbit polyclonal anti-GRIP2—antigen was KLH-conjugated, synthetic peptide (620-649) of human GRIP2; Invitrogen/ThermoFisher #PA5-48489,

Lot XD3568904 (Carlsbad, CA); 0.42 mg/ml diluted with as above to 1:25 (no cross-reactivity with USP53 confirmed by WB; no cross-reactivity with GRIP1 per manufacturer). 3) mouse monoclonal anti-GluA2, IgG1 (UC Davis/NIH NeuroMab Facility, NeuroMab clone L21/32; RRID AB_2877267; 1 mg/ml diluted as above 1:1000).

Secondary antibodies included the following: 1) stabilized peroxidase conjugated goat anti-rabbit (H+L) HRP; Invitrogen/Thermo Fisher Scientific # 32460; stock 10 ug/ml; 1:1000 in TBST; 2) horse HRP-conjugated anti-mouse IgG (H + L) 1:1500 #7076 Cell Signaling Technology (Danvers, MA); 3) [Alexa Fluor 488]-conjugated goat anti-rabbit IgG, cross-absorbed; Thermo Fisher Scientific, Waltham, MA; A32731; 2 µg/ml diluted with PBST, 1% goat serum, 1% BSA); 4) [Alexa Fluor Plus 594]-conjugated goat anti-mouse IgG (H+L), cross-absorbed; Invitrogen/ThermoFisher # A32742; 2 µg/ml diluted as above PBST.

Control immunostaining employed the following: 1) no primary antibody; 2) rabbit polyclonal anti-keyhole limpet hemocyanin; Bethyl Labs A150-104A-7 (Hamburg, Germany); 1 mg/ml diluted 1:200-1,000; 3) antigen-absorbed anti-USP53 (above) with 10x excess human antigen APrEST78787 (beginning at residue 493):

APNGFKQHGNPHLYHSQGKGSYKHDRVVPQSRASA

QIISSSKSQILAPGEKITGKVKSDNGTGYDTDSSQDSRDRGNSCDSSSKSRNR;

Atlas Antibodies, Stockholm Sweden).

Supplementary References

1. Kanwal A, Sheikh SA, Iftikhar A, Naz S, Pardo JV. Preliminary studies on apparent Mendelian psychotic disorders in consanguineous families. *BMC Psychiatry. 2022;* 22, Article number: 709.

2. Grimberg J, Nawoschik S, Belluscio L, McKee R, Turck A, Eisenberg A. A simple and efficient non-organic procedure for the isolation of genomic DNA from blood. *Nucleic Acids Res*. 1989;17(20):8390.

3. Zhu KY, Clark JM. Addition of a competitive primer can dramatically improve the specificity of PCR amplification of specific alleles. *Biotechniques*. 1996;21(4):586.

4. Van der Auwera GA, O'Connor BD. Genomics in the Cloud: Using Docker, GATK, and WDL in Terra (1st Edition). O'Reilly Media. Sebastopol, California: O'Reilly Media; 2020.
